# Hook-and-Destroy Strategy for Efficient Activation of Persulfate by B-Doped Pyrochar for the Removal of Contaminants of Emerging Concern from Wastewater

**DOI:** 10.3390/toxics13121035

**Published:** 2025-11-29

**Authors:** Sanja Panić, Nebojša Vasiljević, Mirjana Petronijević, Igor Antić, Jelena Živančev, Nataša Đurišić-Mladenović

**Affiliations:** 1 Faculty of Technology Novi Sad, University of Novi Sad, Bulevar Cara Lazara 1, 21000 Novi Sad, Serbia; nebojsa.vasiljevic@tfzv.ues.rs.ba (N.V.); mirjana.petronijevic@uns.ac.rs (M.P.); antic@tf.uns.ac.rs (I.A.); jelena.zivancev@tf.uns.ac.rs (J.Ž.); natasa.mladenovic@uns.ac.rs (N.Đ.-M.); 2 Faculty of Technology Zvornik, University of East Sarajevo, 75400 Zvornik, Bosnia and Herzegovina

**Keywords:** metal-free pyrochar catalysts, B-doped pyrochar, persulfate activation, contaminants of emerging concern (CECs), hook-and-destroy strategy, wastewater treatment

## Abstract

This study presents a sustainable and efficient strategy for removing contaminants of emerging concern (CECs) from wastewater using non-metal-doped pyrochar catalysts synthesized via a green, one-step pyrolytic process from pinewood sawdust, urea, and boric acid. The resulting N- and B-doped pyrochars were evaluated for their ability to activate peroxydisulfate (PDS) and degrade a mixture of 25 CECs (15 pesticides and 10 pharmaceuticals). B-doped pyrochar exhibited superior bifunctional performance, combining high adsorption capacity with efficient catalytic PDS activation. Structural characterization confirmed the incorporation of boron into the carbon matrix, generating electron-deficient Lewis acid sites and enhancing the affinity toward PDS and CECs. Quenching and adsorption–degradation analyses revealed a synergistic combination of radical and non-radical pathways, supported by π–π interactions, hydrogen bonding, and Lewis acid–base interactions. Reusability tests confirmed long-term stability and high degradation efficiency over four cycles. These findings demonstrate the potential of B-doped pyrochar as a cost-effective, stable, and environmentally friendly catalyst for practical wastewater treatment.

## 1. Introduction

The availability of clean and safe water is essential for protecting public health and maintaining ecological balance. In recent decades, rapid population growth and intensified human activities have led to the continuous discharge of various chemicals from anthropogenic sources into aquatic environments, posing serious risks to both human health and aquatic ecosystems [1]. Among these pollutants, a group of commonly referred to as contaminants of emerging concern (CECs), includes pharmaceuticals, personal care products, and various industrial chemicals. Many CECs exhibit high mobility through water flows, eventually reaching groundwater and drinking water sources [2]. They are frequently detected in wastewater effluents and even in treated drinking water, yet they remain largely unregulated and are not routinely monitored. Many CECs are persistent and toxic with a potential for bioaccumulation, while their occurrence at trace concentrations limits the efficiency of conventional treatment technologies, underscoring the urgent need for advanced remediation strategies.

As environmental regulations become increasingly stringent, the demand for innovative, efficient, and sustainable treatment technologies is greater than ever. In November 2024, the European Parliament adopted a revised Urban Wastewater Treatment Directive (91/271/EEC), introducing a fourth treatment stage (quaternary treatment) designed to remove at least 80% of selected micropollutants, including some pharmaceuticals and industrial chemicals [3]. In this context, advanced oxidation processes (AOPs) have emerged as promising strategies for the degradation of recalcitrant organic pollutants [4,5]. Among these, persulfate-based AOPs (PS-AOPs) have gained particular attention owing to their strong oxidative potential and cost-effectiveness [6,7]. These processes rely on peroxymonosulfate (PMS) or peroxydisulfate (PDS) to generate reactive oxygen species (ROS), such as sulfate radicals (SO_4_•^−^), hydroxyl radicals (•OH), superoxide radicals (O_2_•^−^), and singlet oxygen (^1^O_2_), or to trigger electron-transfer processes (ETP) [8]. Sulfate radicals, in particular, exhibit a high redox potential (2.5–3.1 V), relatively long half-life (30–40 µs), and stability across a broad pH range (2.0–8.0) [6]. The activation of persulfates typically requires external energy input (e.g., UV, heat, ultrasound) or the use of catalysts [4,8,9,10]. Among these, heterogeneous catalysts stand out due to their simplicity, energy efficiency, and environmental compatibility compared to energy-intensive activation methods [11].

Despite these advantages, the development of high-performance catalysts for persulfate activation remains a major challenge. Effective heterogeneous catalysts must possess optimized physicochemical properties, including tailored band structures, large surface areas, and suitable morphologies, while also complying with the principles of green chemistry. Although transition metal-based catalysts exhibit high catalytic activity, their application is often hindered by drawbacks such as metal leaching, secondary pollution, and poor long-term stability [12].

In this context, carbon-based materials such as biochar, graphene, activated carbon, carbon nanotubes, and nanodiamonds have emerged as promising alternatives for the green remediation of CECs [13,14]. These materials provide large surface areas, abundant functional groups, and intrinsic defects that serve as active sites for both radical and non-radical persulfate activation. Importantly, non-radical pathways, including singlet oxygen generation and electron-transfer processes, offer advantages such as high selectivity, tolerance to background water constituents (e.g., inorganic anions, pH fluctuations), and reduced formation of toxic by-products [15]. Within this group, biochar derived from biomass waste, including pyrochar and hydrochar, stands out as a sustainable and cost-effective alternative to metal-based catalysts. Beyond enabling waste valorization, biochar serves as a highly efficient green catalyst for CEC degradation in PS-AOPs due to its simple preparation, wide availability of feedstocks, and low production cost [10,16]. In particular, pyrochar, obtained through high-temperature pyrolysis of dry biomass under oxygen-limited conditions, contains abundant structural defects, edge sites, and functional groups (e.g., –COOH, –OH, –CO) that promote oxidant activation and facilitate electron shuttling in persulfate-based systems [8,17,18].

However, pristine carbon materials generally suffer from limited intrinsic catalytic activity. To address this limitation, recent studies have focused on their engineering by heteroatom doping to tailor surface properties and enhance catalytic performance. Doping with elements such as B, N, S, Si, Se, and F can modulate the electronic structure, increase defect density, and generate additional active sites [17,19]. For example, nitrogen, with higher electronegativity than carbon (3.04 vs. 2.55), promotes electron transfer and induces charge redistribution, thereby enhancing PMS adsorption on N-doped carbon materials [18,20,21]. Similarly, sulfur, with an electronegativity close to that of carbon (2.58), increases spin density in the lattice, improving conductivity, catalytic activity, and stability [20]. In contrast, boron, with lower electronegativity (2.04) and structural similarity to carbon, forms stable covalent bonds during high-temperature synthesis. B-doping creates electron-deficient Lewis acid sites that promote pollutant adsorption and electron-transfer processes [5,12]. While B-doped carbons have been widely studied in energy-related applications, their role in persulfate-based AOPs remains underexplored. Recent advances increasingly highlight the promise of boron-doped carbon materials as efficient metal-free catalysts for persulfate activation, owing to their ability to introduce electron-deficient Lewis acid sites, enhance charge redistribution, and promote non-radical pathways with high selectivity and stability [5,12]. Compared with conventional carbonaceous materials and pristine biochars, B-doped systems exhibit improved affinity for persulfate and greater electron-transfer capability, enabling faster degradation of diverse organic contaminants [10,17,19]. At the same time, several competing PS-AOP technologies, such as metal-based catalysts, UV-/heat-assisted activation, and Fe-modified biochars, remain limited by issues including metal leaching, secondary pollution, high energy demands, and susceptibility to oxidative deactivation [6,7,8,12]. These recent developments underscore the relevance of designing sustainable, heteroatom-engineered carbocatalysts, particularly those doped with boron, as robust and environmentally compatible alternatives for practical persulfate-based water treatment.

Biochar provides a sustainable and cost-effective platform for the development of B-doped carbocatalysts, as it is derived from renewable waste biomass and can be engineered to achieve high surface area, tunable porosity, and versatile surface chemistry. Moreover, it supports circular economy principles by enabling resource recovery and waste valorization. Engineered biochars, particularly those doped with heteroatoms, are recognized as high-capacity adsorbents for organic pollutants; thus, coupling adsorption with PS-AOPs can significantly enhance the removal efficiency of CECs from wastewater. This integrated hook-and-destroy strategy combines adsorption and catalytic degradation in a synergistic manner: pollutants are first concentrated on the catalyst surface and subsequently degraded [22,23,24]. However, a key limitation of this approach lies in the susceptibility of biochar-based catalysts to radical-induced damage during oxidation. Therefore, the development of stable bifunctional materials with robust adsorption and catalytic performance, capable of withstanding harsh oxidative conditions, is essential for the practical application of PS-AOPs.

Accordingly, this study aims to develop and characterize high-performance, metal-free heteroatom-doped pyrochars for the cooperative adsorption and persulfate oxidation (PSO) of selected CECs in wastewater. Particular emphasis is placed on elucidating the role of boron doping in enhancing pollutant adsorption, facilitating non-radical pathways, and improving catalytic stability under environmentally relevant conditions. By addressing current limitations, this work advances the hook-and-destroy strategy and introduces a novel, green, and efficient B-doped pyrochar catalyst with strong potential for broader application in advanced water treatment technologies.

## 2. Materials and Methods

### 2.1. Chemicals

All chemicals were of analytical grade and used without further purification. Urea (NH_2_CONH_2_, 99.0%, nitrogen source), boric acid (H_3_BO_3_, ≥99.5%, boron source), and potassium persulfate (K_2_S_2_O_8_, ≥99.0%) were purchased from Sigma-Aldrich (St. Louis, MO, USA). Reagents employed in quenching experiments included methanol (CH_3_OH, ≥99.9%), tert-butanol (TBA, ≥99.5%), sodium azide (NaN_3_, ≥99.5%), and chloroform (CHCl_3_, ≥99.5%) (Sigma-Aldrich, St. Louis, MO, USA). Acetonitrile (CH_3_CN, ≥99.9%) (Sigma-Aldrich, St. Louis, MO, USA) was used for the recovery of CECs adsorbed onto catalyst surfaces. Solvents and additives used for sample preparation and instrumental analysis (methanol, acetonitrile, formic acid, ≥99%, and ammonium acetate) were of LC–MS grade (Sigma-Aldrich, St. Louis, MO, USA). Ultrapure water (resistivity 18.2 MΩ·cm, TOC < 5 μg/L) was produced using a Direct-Q^®^ purification system (Millipore, Bedford, MA, USA).

Analytical standards of pesticides and pharmaceuticals were purchased from HPC Standards GmbH (Cunnersdorf, Germany). The pesticide set included acetamiprid (CAS 135410-20-7), carbaryl (CAS 63-25-2), carbofuran (CAS 1563-66-2), diazinon (CAS 333-41-5), dimethoate (CAS 60-51-5), ethoprophos (CAS 13194-48-4), imazalil (CAS 35554-44-0), linuron (CAS 330-55-2), malathion (CAS 121-75-5), methamidophos (CAS 10265-92-6), methidathion (CAS 950-37-8), omethoate (CAS 1113-02-6), phosphamidon (CAS 13171-21-6), propiconazole (CAS 60207-90-1), and trichlorfon (CAS 52-68-6). The pharmaceutical set included acetaminophen (paracetamol, CAS 103-90-2), atenolol (CAS 29122-68-7), carbamazepine (CAS 298-46-4), diltiazem (CAS 42399-41-7), famotidine (CAS 76824-35-6), hydrochlorothiazide (HCTZ, CAS 58-93-5), propranolol (CAS 525-66-6), ranitidine (CAS 66357-59-3), salbutamol (CAS 18559-94-9), and sotalol (CAS 3930-20-9). All standards were ≥98% purity and prepared as individual stock solutions in methanol or acetonitrile. Stock solutions were stored at −20 °C until use.

### 2.2. Preparation of Metal-Free Carbocatalysts

Pinewood sawdust (PW), used as the precursor for pyrochar production, was rinsed, dried, and ground into a fine powder before further processing. The biomass was mixed with urea and/or boric acid at a 1:1 weight ratio, dispersed in water, and ultrasonicated for 30 min. The resulting suspension was subjected to high-energy ball milling at 300 rpm for 15 min using a FRITSCH Planetary Mono Mill Pulverisette 6. The milled material was dried overnight and subsequently annealed in a tube furnace at 800 °C for 2 h under a continuous nitrogen flow (110 mL/min) with a heating rate of 10 °C/min. After thermal treatment, the obtained biochar (BC) was washed with ultrapure water, oven-dried at 80 °C, and stored in a desiccator until use.

The obtained metal-free carbocatalysts were denoted as N-PW-BC, B-PW-BC, and N,B-PW-BC, corresponding to the incorporated dopant(s), N, B, and N simultaneously with B, respectively, in PW-based BC. For comparison, pristine pyrochar (PW-BC) was prepared under identical annealing conditions but without dopants.

### 2.3. Characterization Methods

The morphology and elemental composition of B-PW-BC, identified as the most efficient among the prepared carbocatalysts, were examined using high-resolution scanning electron microscopy (HRSEM, Thermo Fisher Scientific Apreo 2C, Waltham, MA, USA) equipped with energy-dispersive X-ray spectroscopy (EDS). The images were taken at a beam acceleration of 10 kV or 5 kV, a working distance of 10 mm, and a current of 0.10 nA, using the EDT and T1 detectors. The EDX analyses were performed under the same settings. X-ray diffraction (XRD) measurements were performed on a Rigaku Miniflex 600 unit (Rigaku Corporation, Tokyo, Japan) (Cu Kα radiation, λ = 0.15406 nm) using a counting step of 0.3° and a counting time per step of 3 s. Fourier-transform infrared (FTIR) spectra were recorded on a Bruker Vertex 70 spectrometer using the KBr pellet method.

Surface composition and chemical states were analyzed by X-ray photoelectron spectroscopy (XPS, SPECS) equipped with a PHOIBOS 100 energy analyzer (SPECS Surface Nano Analysis GmbH, Berlin, Germany). Measurements were performed in fixed analyzer transmission (FAT) mode with a pass energy of 40 eV and step size of 0.5 eV for survey scans, and 20 eV with 0.1 eV step size for high-resolution spectra. A monochromatic Al Kα source (1486.74 eV, 250 W) was employed under ultra-high vacuum (~5 × 10^−7^ Pa). The sample was mounted on the XPS stage with double-sided conductive adhesive tape. Data processing included Shirley background subtraction and Gaussian–Lorentzian peak fitting using CasaXPS software (version 2.3.26). The textural properties of the B-PW-BC were determined using the method of low-temperature adsorption of N_2_ at the temperature of liquid N_2_ (−196 °C) in the flow of He as the carrier gas (St 1 on NOVA touch 2LX instrument, Quantachrome Instruments, Boynton Beach, FL, USA).

For comparison purposes, HRSEM and XRD analyses were also performed on the pristine pyrochar sample (PW-BC).

### 2.4. Adsorption and Catalytic Oxidation Experiments

Catalytic oxidation experiments with PW-BC, N-PW-BC, B-PW-BC, and N,B-PW-BC were performed in 250 mL beakers containing 100 mL of a single mixture (aqueous model solution) comprising all 25 CECs simultaneously—15 pesticides and 10 pharmaceutically active compounds (PhACs). Two initial concentrations were tested for each compound (10 and 30 μg/L), selected to reflect high levels reported for WWTP influents and effluents in Serbia and the broader Balkan region [25,26,27]. The reactions were carried out at 25 °C. For each experiment, 100 mg of catalyst and 135 mg of potassium persulfate (K_2_S_2_O_8_) were added to the solution, followed by magnetic stirring at 600 rpm. At predetermined time intervals, 1 mL aliquots were withdrawn from the reaction mixture and filtered through a 0.22 μm Nylon syringe filter. A 0.5 mL portion of each filtrate was immediately mixed with 0.5 mL of methanol to quench the reaction. To evaluate adsorption on B-PW-BC, as the most efficient carbocatalyst, experiments were carried out under identical conditions but without persulfate addition.

Residual concentrations of CECs were determined using ultra-high-performance liquid chromatography coupled with triple quadrupole mass spectrometry (UHPLC–MS/MS, Thermo Fisher Scientific, Waltham, MA, USA) without additional sample preparation. Chromatographic separation was achieved on a Rapid Resolution HD ZORBAX Eclipse Plus C18 column (100 × 2.1 mm, 1.8 μm; Agilent Technologies, Santa Clara, CA, USA). Instrumental conditions and optimized parameters for each analyte were reported previously [28]. For quantification, six-point calibration curves were established for all analytes, covering a concentration range from the method limits of quantification (Table S1) to 100 µg/L, which encompassed the spiked sample levels (10 and 30 µg/L). Quality assurance included parallel control samples (non-treated solutions) in each batch to confirm spiked concentrations and verify system performance. All experiments were conducted in duplicate.

In PSO experiments, CECs adsorbed on the surface of B-PW-BC were recovered by ultrasonic treatment of the suspension in acetonitrile for 20 s, and the supernatant was analyzed by UHPLC–MS/MS. The used recovery method was adopted from Chu et al. with slight modifications [22]. The same carbocatalyst was tested on a real wastewater effluent sample from a local WWTP, which was filtered through a 0.45 μm glass-fiber membrane filter before PSO. The measured characteristics of the wastewater sample after the filtration were: UV_254_ = 0.124, turbidity 0.57 NTU, pH = 8, and TOC = 8.15 mgC/L. After PSO, a wastewater sample was prepared using solid-phase extraction (SPE) with Oasis HLB cartridges (6 mL, 200 mg; Waters Corp., Milford, MA, USA). The detailed sample preparation procedure was described in [28].

### 2.5. Catalyst Stability and Reusability

The stability and reusability of the B-PW-BC catalyst were evaluated through four consecutive catalytic oxidation cycles. After each run, the catalyst was collected by filtration, thoroughly washed with ultrapure water to remove residual reactants and by-products, oven-dried at 80 °C, and reused under identical experimental conditions. The residual catalytic performance was expressed in terms of CECs removal efficiency after each cycle.

### 2.6. Quenching Experiments

To elucidate the reactive oxygen species (ROS) involved in CEC degradation, quenching experiments were conducted under the same conditions as the catalytic oxidation tests, with the addition of specific scavengers. A persulfate-to-scavenger molar ratio of 1.7 was applied, following literature recommendations [29]. Methanol was used to quench both •OH and SO_4_•^−^, whereas tert-butanol (TBA) selectively scavenged •OH [17]. Chloroform was employed as an O_2_•^−^ scavenger, and sodium azide (NaN_3_) was used to quench ^1^O_2_.

## 3. Results and Discussion

### 3.1. PDS Catalytic Activity of Metal-Free Pyrochar-Based Materials

**Figure 1:**
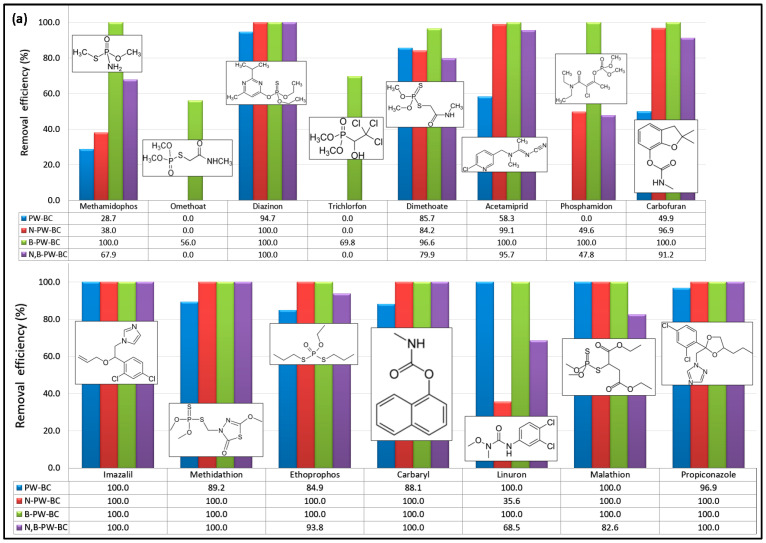

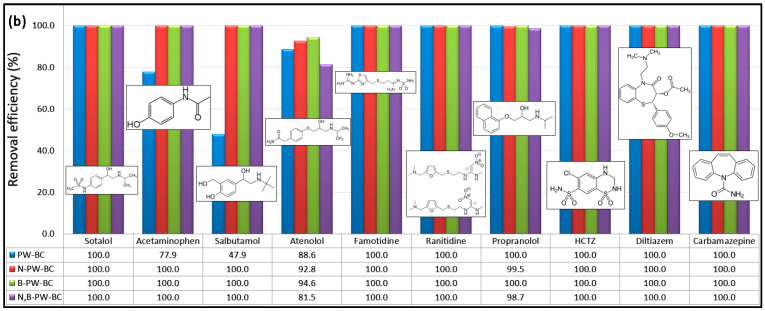
Removal efficiencies (%) of selected (**a**) pesticides and (**b**) PhACs in the presence of metal-free pyrochar-based catalysts after 90 min of oxidation process (experimental conditions: [individual CEC] = 10 μg/L; [carbocatalyst] = 1 g/L; [PDS] = 5 mM; T = 25 °C; initial solution pH = 6.5). An efficiency of 100% was achieved when the residual concentration of the compound of interest at the end of the process was below the quantification limit of the applied method.

The catalytic performance of the synthesized metal-free carbocatalysts was evaluated in the presence of PDS using a model aqueous solution containing 15 pesticides and 10 PhACs (10 μg/L each). Figure 1 shows the removal efficiencies of the selected CECs obtained for four different catalytic systems after 90 min of reaction.

As shown in Figure 1, all tested carbocatalysts displayed higher reactivity toward PhACs compared to pesticides. Notably, omethoate and trichlorfon were oxidized only in the presence of the B-doped sample, whereas pristine pyrochar was completely inactive for phosphamidon degradation. Pristine pyrochar showed modest activity (<60% removal) toward methamidophos, acetamiprid, and carbofuran. The N-doped pyrochar achieved <50% removal of methamidophos, phosphamidon, and linuron, while it exhibited excellent activity toward dimethoate (84.2%) and nine other pesticides that were nearly completely degraded within 90 min. In contrast, the dual-doped material (N,B-PW-BC) did not show consistent enhancement compared to the N-doped catalyst: improvements were observed for methamidophos and linuron, but reduced activity was noted for carbofuran, ethoprophos, and malathion. Overall, only six pesticides (diazinon, acetamiprid, imazalil, methidathion, carbaryl, and propiconazole) were almost completely removed (>95%) with the N,B-codoped pyrochar after 90 min. In comparison, the B-doped pyrochar exhibited the most pronounced catalytic effect. In combination with PDS, it achieved near-complete removal (>95%) of 13 out of the 15 pesticides tested, confirming that boron incorporation substantially enhanced the PDS activation capacity of pyrochar.

The removal efficiencies of PhACs highlighted the strong catalytic potential of heteroatom-doped pyrochars (Figure 1b). Pristine pyrochar showed the lowest activity toward salbutamol and only moderate performance for acetaminophen and atenolol. The N-doped pyrochar exhibited high catalytic activity, achieving nearly complete removal (~100%) of nine PhACs, with only a slight reduction in efficiency for atenolol. In contrast, codoping with N and B did not provide additional benefits compared with single-doped systems. The B-doped pyrochar demonstrated the best overall performance, achieving almost complete degradation of all PhACs tested, with removal efficiencies close to 100% for ten compounds and 94.5% for atenolol. Overall, all tested carbocatalysts showed substantially higher affinity toward PhACs compared to pesticides, as seven PhACs were almost completely removed across all catalytic systems. These findings confirm that heteroatom incorporation into the carbon framework significantly enhances the PDS activation performance of pyrochar-based catalysts.

**Figure 2:**
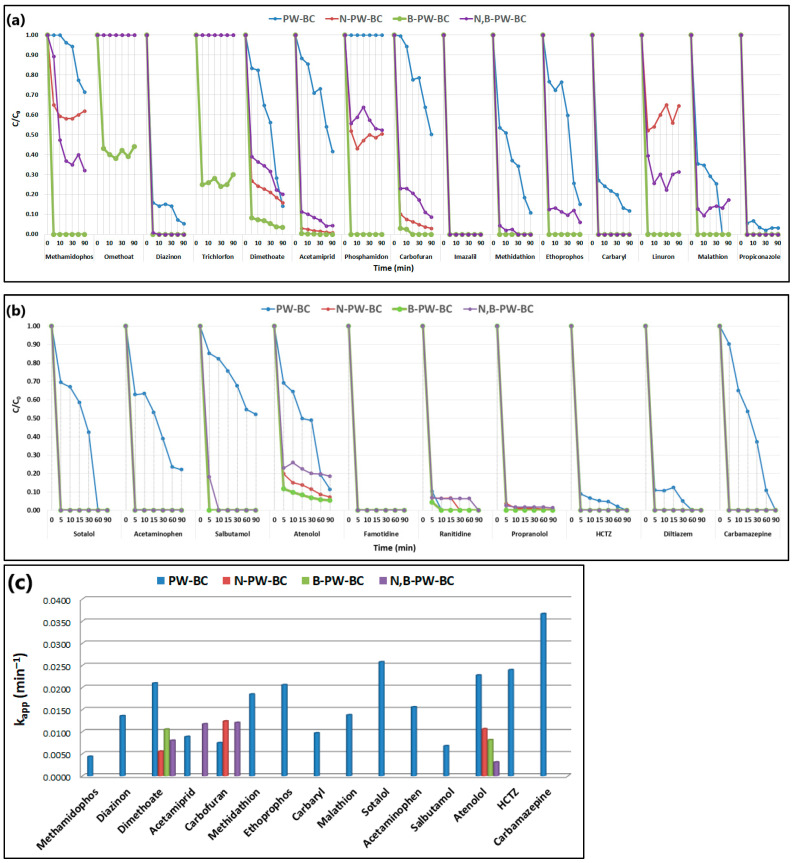
Degradation kinetics of selected CECs ((**a**) pesticides, (**b**) PhACs) in metal-free pyrochar-based catalysts/PDS system with (**c**) corresponding values of the pseudo first-order rate constant.

The catalytic performance of pristine and heteroatom-doped pyrochars was further evaluated by fitting the experimental data to a pseudo-first-order kinetic model (Figure 2):

$$C=C_{0}e^{-k_{app}t}$$

where C and C_0_ represent the pollutant concentrations at time t and 0 min, respectively, and k_app_ is the apparent rate constant.

In the presence of pristine pyrochar, six pesticides (methamidophos, dimethoate, acetamiprid, carbofuran, methidathion, and ethoprophos) showed relatively consistent degradation with the highest rate constant for dimethoate (k_app_ = 0.021 min^−1^). Diazinon, carbaryl, malathion, and propiconazole exhibited a rapid initial removal (>60% within 5 min), followed by slower degradation, while carbaryl showed the lowest rate constant (0.0097 min^−1^). Imazalil and linuron were completely removed within 5 min. N-doped pyrochar accelerated pesticide degradation, with most compounds fully removed after 5 min, except dimethoate and carbofuran, which degraded more slowly (k_app_ = 0.0056 and 0.0124 min^−1^, respectively). The N,B-codoped system showed a similar trend, achieving >70% removal of nine pesticides within 5 min but with slower subsequent degradation of dimethoate, acetamiprid, and carbofuran. By contrast, B-doped pyrochar demonstrated exceptional catalytic activity, achieving >90% removal of 13 pesticides within 5 min, while omethoate and trichlorfon were degraded only during the initial stage of the process. Regarding the PhACs, distinct kinetic profiles were observed with pristine pyrochar, particularly for sotalol, acetaminophen, salbutamol, atenolol, and carbamazepine, the latter showing the highest rate constant (k_app_ = 0.0367 min^−1^). The incorporation of heteroatoms (N, B, or both) markedly enhanced performance, resulting in rapid degradation of all PhACs, with most compounds almost completely removed within 5 min. Both single-doped and codoped pyrochars significantly outperformed pristine pyrochar, with the B-doped catalyst again showing the highest overall activity.

The dual N,B-doped pyrochar did not exhibit a consistent improvement in catalytic activity compared to single-doped materials, suggesting that the simultaneous introduction of these two heteroatoms does not necessarily lead to the expected synergistic enhancement. One possible explanation is that concurrent incorporation of nitrogen and boron can generate competitive or even antagonistic effects on the electronic structure of the carbon matrix [12,13]. Nitrogen, being more electronegative than carbon, enriches adjacent sites with electron density and typically facilitates radical-type persulfate activation pathways, whereas boron introduces electron-deficient Lewis acid centers that promote non-radical mechanisms such as direct electron transfer or surface-bound reactive complexes [5,9]. When these dopants coexist within the same local domains, their opposite electronic influences may partially counterbalance one another, diminishing the net charge polarization and thereby lowering the density of catalytically relevant active sites. Moreover, codoping can perturb the structural integrity of the carbon lattice more severely than single-atom substitution. The introduction of heteroatoms with different atomic radii and bonding preferences may generate nonuniform defect clusters, distort graphitic domains, or interrupt π-electron delocalization, leading to suboptimal defect distribution and reduced electronic conductivity [13]. Such structural perturbations can also decrease accessible porosity or block functional groups that are otherwise available in single-doped materials, ultimately hindering the adsorption of persulfate and target CECs. Similar observations have been reported for other multi-heteroatom-doped carbons, where the expected synergistic effects fail to occur due to over-doping or lattice disorder [20]. Taken together, these electronic and structural interferences provide a plausible explanation for the weaker-than-anticipated catalytic performance of the N,B-codoped pyrochar, suggesting that in this system dual doping induced competition rather than cooperation between active sites.

For broader context and to strengthen the performance assessment, it is important to note that the catalytic behavior of the synthesized pyrochars is comparable to, and in several cases surpasses, that reported for commercial activated carbon and other established carbon-based catalysts used for persulfate activation. Previous studies have shown that activated carbon and conventional metal-free carbons typically exhibit moderate removal efficiencies and often rely predominantly on radical pathways with limited selectivity and susceptibility to surface oxidation [10,17]. In contrast, the B-doped pyrochar developed in this study demonstrated markedly faster degradation kinetics and broader reactivity toward structurally diverse CECs. Similar improvements have been reported for heteroatom-engineered carbons, where dopants such as N or B increase defect density and promote non-radical electron-transfer pathways, resulting in higher stability and more efficient PDS activation than commercial activated carbon [12,19]. These comparative trends indicate that the performance of the B-doped pyrochar is fully aligned with, and in many cases superior to, benchmark carbonaceous catalysts commonly applied in PS-AOPs.

### 3.2. Characterization of B-Doped Carbocatalyst

**Figure 3:**
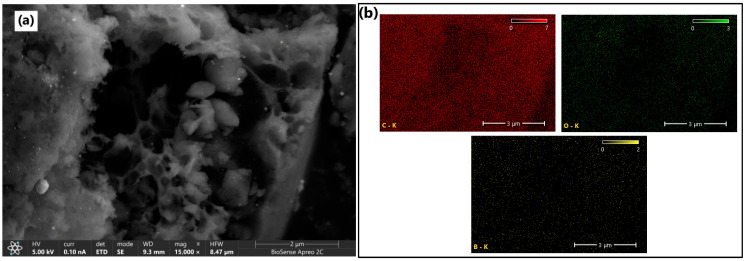
(**a**) HRSEM image of B-doped pyrochar with (**b**) corresponding element mapping for C, O, and B determined by EDX analysis.

The surface morphology of the synthesized B-PW-BC catalyst was examined by HRSEM, while elemental composition and distribution were determined by EDX analysis. As shown in Figure 3a, the material exhibited a heterogeneous structure with smooth surfaces and well-developed macropores. Certain structural features resembled the lignocellulosic cell wall architecture of the pinewood precursor, appearing as channel-like units with sharp edges. The abundant porous structure can be attributed to the presence of boric acid during pyrolysis, which generates additional gas release [30], and catalyzes dehydration and depolymerization reactions of the biomass, thereby promoting porosity development [31]. EDX mapping (Figure 3b) confirmed the presence of carbon (81.0 at.%), oxygen (8.9 at.%), and boron (10.1 at.%) with uniform spatial distribution, indicating the successful incorporation of boron into the carbon framework. To highlight structural differences between B-doped and undoped samples, HRSEM analysis was also performed on the pristine pyrochar (Figure S1), confirming the pore development and changes in surface texture induced by boron incorporation. The pristine pyrochar exhibited an irregular and heterogeneous lamellar structure with fractured sheet-like domains. The surface morphology shows compact regions with limited pore development, suggesting predominantly macroporous characteristics of this sample. Some surface particles were also observed, likely corresponding to mineral residues inherited from the inorganic constituents of the pinewood.

The adsorption–desorption isotherm of the B-PW-BC sample exhibits an H4-type hysteresis loop, indicating the presence of a certain proportion of micropores. The Brunauer–Emmett–Teller (BET) surface area is 81.5 m^2^/g. The pores are distributed within the range of up to 10 nm in diameter, with a total pore volume of 0.015 cm^3^/g and an average pore diameter of 2.2 nm. Therefore, the porous structure of B-PW-BC is predominantly microporous, with a fraction of pores falling within the mesoporous domain and extending toward the microporous range.

**Figure 4:**
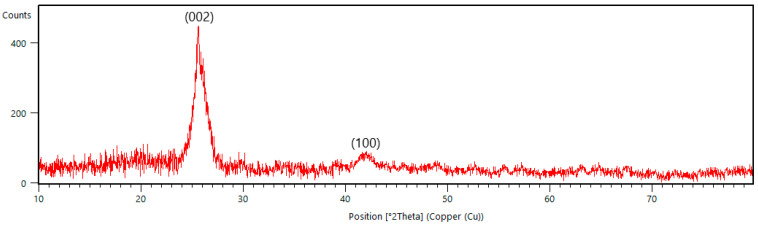
XRD pattern of B-doped pyrochar.

XRD analysis of the B-PW-BC catalyst provided insight into its crystal structure (Figure 4). During pyrolysis, the decomposition of cellulose, hemicellulose, and lignin in pinewood biomass generates free monomeric units, which condense into amorphous carbonaceous material composed of aliphatic chains and aromatic rings. Progressive condensation of these aromatic units leads to the formation of conjugated sheets that organize into a turbostratic carbonaceous structure [32]. The XRD pattern of B-PW-BC displayed two characteristic peaks of turbostratic carbon: the C(002) reflection at 2θ = 25.8° and the C(100) reflection at 2θ = 42.1° (PCPDFWIN database, CAS 75–1621). The relatively high intensity of the (002) peak indicates the presence of a turbostratic phase with a considerable degree of crystallinity, while the (100) peak is associated with lateral growth of carbon domains through linking of aromatic sheets [33]. Compared with the pristine pyrochar (Figure S2), the peak positions for these two peaks in B-PW-BC catalyst were shifted by 1.4° (002) and 0.8° (100) to smaller angles due to lattice distortion and defect introduction. Differences in crystallite stacking among these samples indicate that boron insertion modifies turbostratic carbon ordering.

**Figure 5:**
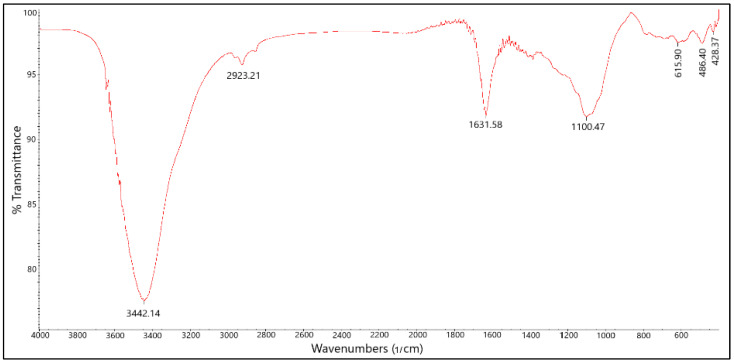
FTIR spectra of B-doped pyrochar.

FTIR spectroscopy was employed to investigate the surface functional groups of the B-PW-BC catalyst (Figure 5). The spectrum exhibited a broad, intense band at 3442 cm^−1^, attributed to –OH stretching vibrations. Aliphatic –CH_2_ (asymmetric) and –CH_3_ (symmetric) stretching vibrations were observed in the range of 2800–3000 cm^−1^. A band at 1631 cm^−1^ was assigned to aromatic –C=C– stretching, as well as –OH vibrations from physisorbed water molecules. Importantly, a broad absorption band around 1100 cm^−1^, corresponding to vibrations of C–B, B–O, and/or C–O bonds, confirmed the successful incorporation of boron atoms into the carbon framework of the pyrochar [12].

**Figure 6:**
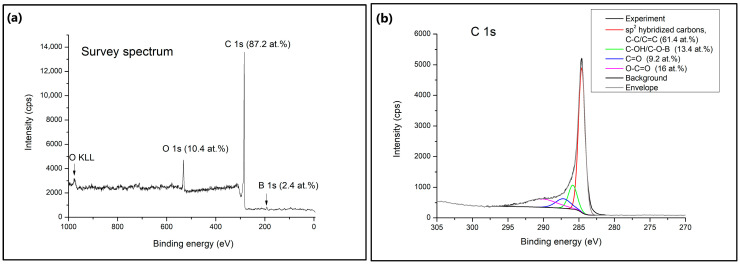

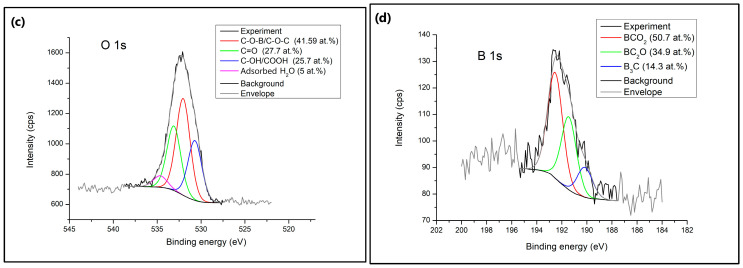
(**a**) XPS survey spectrum with the high-resolution XPS spectrum for (**b**) C 1s, (**c**) O 1s, and (**d**) B 1s of B-doped pyrochar.

X-ray photoelectron spectroscopy (XPS) was used to identify the surface chemical composition of the B-PW-BC sample. As can be seen from the survey spectrum (Figure 6a), the examined sample is composed of three elements, C (87.2 at.%), O (10.4 at.%), and B (2.4 at.%). The C 1s spectra exhibited a highly intense sharp peak which could be deconvoluted into four main peaks at 284.6 eV, 285.9 eV, 287.3 eV and 290.1 eV, assigned to sp^2^ hybridized carbons (C–C/C=C) (61.4 at.%), C–OH/C–O–B (13.4 at.%), C=O (9.2 at.%) and O–C=O (16 at.%), respectively. Three types of oxygen-containing species were registered in O 1s spectra at 530.7 eV, 532.1 eV, and 533.2 eV, demonstrating the structures C–OH/COOH (25.7 at.%), C–O–B/C–O–C (41.6 at.%), and C=O (27.7 at.%), respectively. The obtained signals matched for B 1s are identified as B_3_C at 190.2 eV, BC_2_O at 191.5 eV, and BCO_2_ at 192.6 eV. The highest portion of boron species was introduced into the carbon lattice as BCO_2_ moieties (50.7 at.%), followed by BC_2_O (34.9 at.%) and B_3_C (14.3 at.%). The charge distribution of the carbon network can be regulated by boron doping due to its lower electronegativity (2.04), compared to C (2.55), and electron-deficient characteristics [17]. Also, boron atoms act as Lewis acid sites for enhanced adsorption of organic pollutants, subsequently promoting their cooperative degradation by adsorption and oxidation [12]. BCO_2_ moieties, as the most common ones in the B-PW-BC sample, were formed by boron replacing carbon atoms at the trigonal sites. These groups could affect the electronic behavior of the carbon plane, boosting the oxidation activity of the carbon atoms [34]. The boron content obtained by the XPS method is much lower compared to the one obtained by the EDX, since these methods operate on different principles. XPS is a surface-sensitive technique performed on the top few nanometers of a sample, while EDX is a bulk analysis technique that provides information about the overall elemental composition of a larger volume of the sample. Therefore, the amount of boron present on the surface of the B-PW-BC sample is much smaller compared to its amount in the interior, while slightly higher amount of surface oxygen is due to the presence of oxygen-containing functional groups.

### 3.3. Adsorption Behavior and PDS Catalytic Activity of B-Doped Carbocatalyst

Adsorption plays a crucial role in heterogeneous catalysis, particularly with carbon-based materials that provide high surface area and abundant adsorption sites for organic pollutants [35]. To evaluate this contribution, adsorption experiments (without persulfate) were conducted prior to PDS activation using the B-doped pyrochar, identified as the most efficient among the prepared carbocatalysts. Although the experiments were performed for 90 min, equilibrium was reached within the first 5 min.

**Figure 7:**
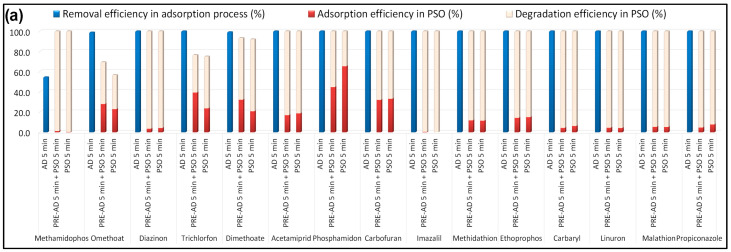

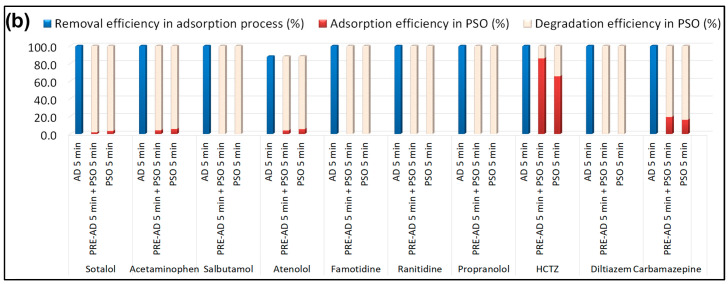
Removal efficiencies (%) of selected (**a**) pesticides and (**b**) PhACs in the presence of B-doped pyrochar after 5 min of adsorption; 5 min of pre-adsorption and 5 min of PSO; and 5 min of PSO (experimental conditions: [individual CEC] = 10 μg/L; [carbocatalyst] = 1 g/L; [PDS] = 5 mM; T = 25 °C; initial solution pH = 6.5). An efficiency of 100% was achieved when the residual concentration of the compound of interest at the end of the process was below the quantification limit of the applied method.

As shown in Figure 2a,b, the combination of B-doped pyrochar with PDS resulted in rapid and nearly complete removal of all targeted pollutants within 5 min. To further examine the cooperative effect of adsorption and oxidation, experiments were performed with and without a pre-adsorption step. After each run, residual CECs were extracted from the catalyst surface to quantify the fraction removed by adsorption versus oxidative degradation (Figure 7).

**Figure 8:**
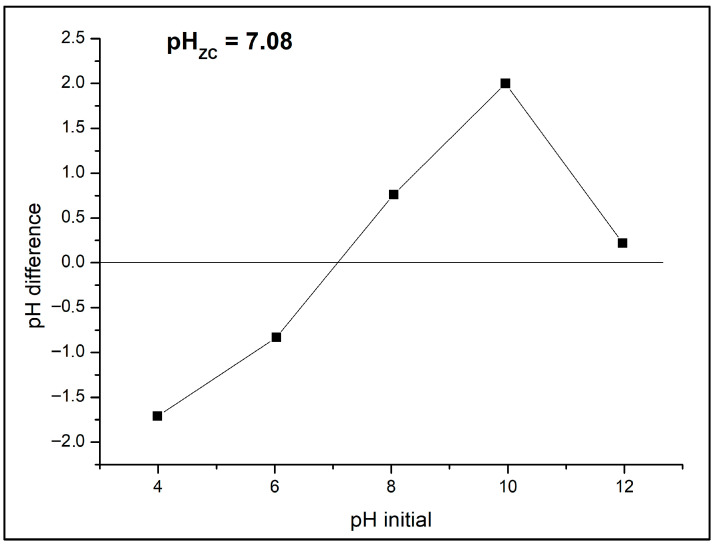
Point of zero charge of B-doped pyrochar.

The point of zero charge (PZC) is a key parameter governing the adsorption of charged molecules on pyrochar surfaces. For the B-doped pyrochar, the measured pH was 6.98, while the PZC determined by the pH drift method was 7.08 (Figure 8). Since the PZC value is very close to the initial solution pH of the selected CECs, the catalyst surface can be considered predominantly neutral under the experimental conditions. This neutrality favors both adsorption and subsequent catalytic degradation by minimizing electrostatic repulsion effects. The abrupt drop of pH difference at basic pH is the consequence of rapid hydrolysis of boron-containing surface groups and surface deprotonation of –OH groups. Both processes generate negatively charged species that promote proton uptake from solution, leading to a sudden decrease in pH during pH drift measurements. Similar behavior has been reported in boron-modified biochars and carbon materials [5,12].

As shown in Figure 7, B-doped pyrochar exhibited a high adsorption capacity toward almost all tested CECs (15 pesticides and 10 PhACs) with diverse chemical functionalities. Only methamidophos (54.5%) among pesticides and atenolol (88.1%) among PhACs showed lower removal efficiencies. The exceptionally high adsorption potential of B-PW-BC can be attributed to its developed porous structure, which provides abundant and easily accessible adsorption sites. To evaluate possible driving forces, the hydrophobicity and ionization properties of the target CECs were considered. The investigated compounds cover a wide range of logK_ow_ values (pesticides: −0.8 for methamidophos to 3.81 for diazinon; PhACs: −0.16 for famotidine to 3.48 for propranolol). The uniformly high adsorption observed in this study cross the wide range of logK_ow_ values of the investigated compounds indicates that hydrophobic interactions played only a minor role [36]. Similarly, electrostatic interactions are unlikely: the catalyst surface was nearly neutral (pH_ZC_ = 7.08), pesticides displayed varied pKa values (mostly > pH of the solution), and PhACs were predominantly neutral under the tested conditions [37]. The absence of clear correlations with logK_ow_ or pKa confirms that neither hydrophobic partitioning nor electrostatics were the dominant mechanisms. Instead, the adsorption of CECs was primarily governed by strong hydrogen bonding and π–π/n–π interactions. Oxygen- and nitrogen-containing groups (–OH, COOH, C=O, pyridinic-N, pyrrolic-N, and amino-N) on the pyrochar surface served as H-bond donors/acceptors for polar functional groups within the pollutant molecules [38]. Aromatic structures in eight pesticides and all PhACs enabled π–π donor–acceptor interactions with the sp^2^-hybridized carbon domains of the pyrochar [39], further reinforced in compounds containing fused heteroaromatic rings [17]. In addition, n–π interactions between surface oxygen functionalities of the catalyst and aromatic rings of CECs likely contributed to adsorption [40]. Importantly, boron species incorporated into the carbon lattice introduced Lewis acid sites, enhancing surface affinity for pollutants and acting as potential catalytic centers for subsequent oxidation [41].

### 3.4. Cooperative Adsorption and Catalytic Oxidation

The catalytic performance of B-doped pyrochar was further investigated by coupling with PDS, with and without a pre-adsorption step. Based on mass balance, CECs in the initial solution were divided into three fractions: physically adsorbed, dissolved, and degraded. The total removal efficiency of each compound (Figure 7) was calculated as the sum of adsorbed and degraded fractions.

B-PW-BC exhibited excellent catalytic performance toward seven pesticides (methamidophos, diazinon, imazalil, carbaryl, linuron, malathion, and propiconazole), achieving nearly complete degradation within 5 min of PDS addition, regardless of pre-adsorption. For trichlorfon and dimethoate, simultaneous adsorption and oxidation resulted in higher degradation (51.4% and 71.2%, respectively) compared to the two-step process (37.2% and 61.2%). In contrast, omethoate and phosphamidon were more efficiently oxidized when pre-adsorption was applied (41.4% and 55.2%) relative to the simultaneous process (33.7% and 34.6%). Several other pesticides (dimethoate, acetamiprid, phosphamidon, carbofuran, methidathion, and ethoprophos) were efficiently removed, but a significant fraction remained adsorbed on the catalyst.

As shown in Figure 7b, the B-doped catalyst promoted rapid and nearly complete degradation of salbutamol, famotidine, ranitidine, propranolol, and diltiazem. Sotalol and acetaminophen reached ≥95% degradation, while atenolol and carbamazepine were degraded by >80%, though a considerable fraction of carbamazepine remained adsorbed. Hydrochlorothiazide (HCTZ) exhibited the lowest reactivity, with degradation efficiencies of 14.2% (pre-adsorption step) and 34.4% (simultaneous process), indicating adsorption-dominant removal.

To further assess the cooperative effect, additional experiments were performed at 30 μg/L per compound. Adsorption equilibrium was achieved within 10 min, after which most pesticides, except methamidophos and omethoate, were almost completely removed by adsorption. Subsequent catalytic oxidation experiments (10 and 90 min) revealed similar removal trends to those observed at lower concentrations, confirming the efficiency and robustness of the hook-and-destroy mechanism.

These results confirm that B-doped pyrochar effectively integrates adsorption and catalytic oxidation, with the balance between adsorbed and degraded fractions depending on pollutant type and reaction mode. The cooperative hook-and-destroy effect was particularly evident for pesticides such as trichlorfon and dimethoate, while for recalcitrant compounds (e.g., HCTZ), adsorption dominated removal.

The data in Table 1 demonstrate that increasing the initial concentration enhanced the adsorption capacity of B-doped pyrochar for most pesticides. Upon PDS addition, methamidophos was almost completely degraded within 10 min, highlighting its high susceptibility to oxidation. Extending the reaction time to 90 min markedly improved degradation efficiencies for several compounds, including diazinon (>84%), linuron (>75%), and malathion (≈100%), irrespective of pre-adsorption. For imazalil (84.7%), methidathion (70.4%), ethoprophos (81.0%), and propiconazole (89.0%), extended treatment was effective only when combined with a pre-adsorption step.

A clear cooperative effect of simultaneous adsorption and oxidation was observed for phosphamidon (55.0%) and carbofuran (76.1%), which showed substantially higher degradation within 10 min compared to the sequential process. By contrast, for omethoate, diazinon, trichlorfon, dimethoate, acetamiprid, ethoprophos, carbaryl, linuron, and malathion, the pre-adsorption step did not significantly affect catalytic performance. Interestingly, a negative impact of pre-adsorption was noted for methidathion and propiconazole, where the highest degradation levels (70.4% and 89.0%, respectively) were achieved after 90 min of oxidation without prior adsorption.

**Figure 9:**
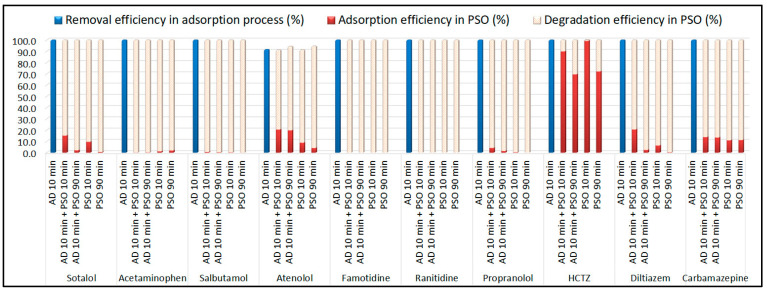
Removal efficiencies (%) of selected PhACs in the presence of B-doped pyrochar after 10 min of adsorption, 10 min of pre-adsorption, and 10 and 90 min of PSO, and 10 and 90 min of PSO (experimental conditions: [individual CEC] = 30 μg/L; [carbocatalyst] = 1 g/L; [PDS] = 5 mM; T = 25 °C; initial solution pH = 6.5). An efficiency of 100% was achieved when the residual concentration of the compound of interest at the end of the process was below the quantification limit of the applied method.

The B-doped pyrochar exhibited outstanding adsorption affinity toward all selected PhACs, achieving nearly complete removal (~100%) for nine out of ten compounds (Figure 9). Beyond its superior adsorption, the material also displayed high catalytic activity for PDS activation. After 90 min of simultaneous adsorption and oxidation, seven PhACs (sotalol, acetaminophen, salbutamol, famotidine, ranitidine, propranolol, and diltiazem) were almost completely degraded.

For atenolol and carbamazepine, a small fraction of the pollutants remained physically adsorbed on the catalyst surface, with degradation efficiencies of 90.4% and 88.7%, respectively, in the absence of a pre-adsorption step. In contrast, hydrochlorothiazide (HCTZ) was removed predominantly through adsorption, with only ~30% oxidized, regardless of prior adsorption.

Overall, these results confirm the cooperative benefit of simultaneous adsorption and persulfate-driven oxidation. The synthesized B-doped pyrochar therefore functions as a bifunctional material, integrating both adsorption sites and catalytic centers, and enabling adsorption-enhanced degradation of PhACs.

### 3.5. The Plausible Mechanism of PDS Activation—Quenching Experiments

To identify the reactive oxygen species (ROS) responsible for CEC degradation, scavenging tests were performed with four common quenchers at a persulfate-to-scavenger molar ratio of 1.7 [29]. All experiments were conducted as simultaneous adsorption–oxidation processes (without pre-adsorption) at an initial concentration of 30 μg/L per compound.

For the majority of tested pollutants, no significant differences in removal efficiencies were observed in the presence of scavengers compared to control runs. Consequently, most CECs, including 11 pesticides (e.g., diazinon, dimethoate, carbofuran, malathion, propiconazole) and 10 PhACs (e.g., sotalol, propranolol, HCTZ, carbamazepine), were almost completely degraded within the first 5 min of reaction in all cases, indicating that the applied quenchers did not exert a clear inhibitory effect.

**Figure 10:**
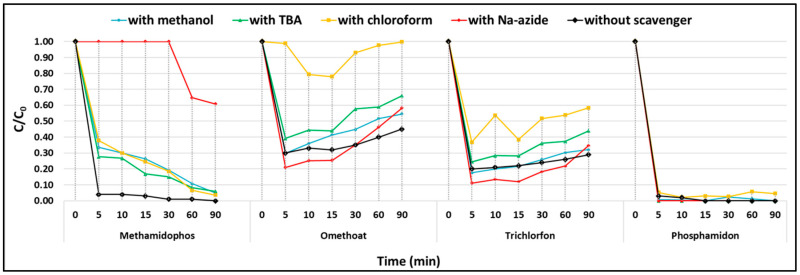
Effects of various scavengers on removal efficiencies of methamidophos, omethoate, trichlorfon, and phosphamidon (experimental conditions: [individual CEC] = 30 μg/L; [carbocatalyst] = 1 g/L; [PDS] = 5 mM; T = 25 °C; initial solution pH = 6.5; PS:scavenger molar ratio 1.7).

Notable exceptions were methamidophos, omethoate, trichlorfon, and phosphamidon, where distinct changes in degradation profiles were recorded in the presence of the respective scavengers (Figure 10). These results suggest that ROS participation in the degradation pathway is compound-specific and more evident for certain pesticides, while for the majority of CECs, adsorption combined with fast electron-transfer reactions dominated the overall removal.

**Radical pathways.** PDS can be activated in metal-free systems through both radical and non-radical processes. Methanol (•OH and SO_4_•^−^ scavenger) and tert-butanol (selective for •OH) were used to probe radical species. In the presence of these quenchers, methamidophos removal was significantly suppressed (from 96.3% to 66.4% with MeOH and 72.4% with TBA), confirming the involvement of both •OH and SO_4_•^−^. Minor inhibition was observed for omethoate and trichlorfon with TBA, though this may partly reflect hydrophobic adsorption of TBA onto the catalyst surface, limiting persulfate access rather than selectively quenching •OH [42].

**Role of superoxide and singlet oxygen.** Chloroform addition indicated the contribution of O_2_•^−^, particularly in the degradation of trichlorfon (efficiency reduced from 79.4% to 63.2%) and methamidophos (96.3% to 62.1%). Omethoate degradation was almost completely suppressed, suggesting a vital role of O_2_•^−^ in its decomposition. Sodium azide (NaN_3_) was used to probe singlet oxygen (^1^O_2_). While its effect was negligible for phosphamidon and trichlorfon, methamidophos degradation was strongly suppressed (only 39.1% removed after 30 min), confirming that ^1^O_2_ played a decisive role. ^1^O_2_ originates from the breakage of the peroxide bond, and typically, can be generated from photoexcitation of oxygen molecules, recombination of O_2_•^−^, and PDS activation by the ketonic group of the carbonaceous catalyst [12,43,44]. The generation of ^1^O_2_ in the examined system is consistent with the presence of C=O functionalities on the catalyst surface (identified by XPS, Figure 6), which can facilitate electron transfer with persulfate [43].

**Non-radical electron transfer pathway (ETP).** For most CECs (11 pesticides—diazinon, dimethoate, acetamiprid, carbofuran, imazalil, methidathion, ethoprophos, carbaryl, linuron, malathion, propiconazole and 10 PhACs—sotalol, acetaminophen, salbutamol, atenolol, famotidine, ranitidine, propranolol, HCTZ, diltiazem, carbamazepine), degradation efficiencies were unaffected by scavengers, despite near-complete removal within minutes. This suggests that a non-radical electron transfer pathway was dominant. In this mechanism, the catalyst acts as a conductor mediating electron transfer between adsorbed organics and persulfate. Depending on substrate properties, two pathways can operate: (i) electron shuttle, favored by electron-rich compounds, and (ii) adjacent transfer, where electron-poor organics capture electrons from adsorbed persulfate [22]. The electron shuttle mechanism could be related to carbofuran and carbaryl, having the highest overall electron richness compared to other 9 pesticides. In the case of 10 PhACs, the degradation of acetaminophen, salbutamol, and propranolol, as electron-rich compounds, could follow the same type of ETP, while the adjacent transfer might be more typical for the rest of 16 electron-poor CECs. Co-adsorption of CECs and persulfate on the catalyst surface is essential for ETP to create ternary mediators (electron acceptor-conductor-electron donor), which would further trigger the electron transfer [45,46]. Hence, to improve the contribution of ETP in the degradation of CECs, it is of great importance to design and synthesize a carbocatalyst with synergistic adsorptive and catalytic functionality.

**Contribution of B doping.** Boron incorporation (BCO_2_, BC_2_O, B_3_C species) creates Lewis acid sites that facilitate pollutant adsorption and persulfate activation. The substitution of C by B lowers electronegativity (2.04 vs. 2.55), redistributes charge density, and introduces structural defects, all of which enhance electron-transfer reactivity [41,47]. Consequently, B-doped pyrochar provides synergistic adsorption and catalytic centers, accelerating pollutant degradation and continuously regenerating free sites for re-adsorption.

**Overall mechanism.** The degradation of organophosphorus pesticides (e.g., methamidophos, omethoate, trichlorfon, phosphamidon) proceeds via combined radical (•OH, SO_4_•^−^, O_2_•^−^) and non-radical (^1^O_2_, ETP) pathways, consistent with their diverse electron-withdrawing and electron-donating substituents. For most other pesticides and all PhACs, non-radical ETP dominated, facilitated by π–π interactions, hydrogen bonding, and boron-induced Lewis acidity. These findings highlight the bifunctional role of B-doped pyrochar in the hook-and-destroy strategy, where pollutants are first adsorbed and subsequently oxidized through synergistic radical and non-radical mechanisms.

### 3.6. Stability of B-Doped Pyrochar—Reusability Test

Industrial catalytic processes require stable catalysts that can withstand prolonged operation or multiple batch cycles [29]. Within the hook-and-destroy strategy, however, catalyst deactivation can occur due to surface oxidation of the carbon lattice during pollutant degradation, particularly via radical pathways [22,48]. Literature reports suggest that boron incorporation can mitigate this effect, as strong C–B bonds suppress carbon lattice oxidation and thereby enhance long-term durability of carbocatalysts [49].

To assess the stability of B-doped pyrochar, reusability experiments were carried out for the degradation of 25 selected CECs over four consecutive adsorption–oxidation cycles (Table 2). After each cycle, the catalyst was recovered, washed, and reused under identical conditions.

The reusability of B-doped pyrochar was confirmed by its consistently high performance in removing all tested CECs across four consecutive cycles. Only minor declines in efficiency were observed for methamidophos (100% → 98.2%), omethoate (49.7% → 39.1%), trichlorfon (70.4% → 62.9%), and atenolol (95.6% → 93.9%). This slight deactivation can be attributed to their degradation pathways, which, as indicated by quenching tests, involve both radical and ^1^O_2_-mediated non-radical processes—pathways more prone to partial catalyst oxidation.

For all other pesticides and PhACs, the B-doped pyrochar retained nearly identical removal efficiencies even after four cycles, demonstrating excellent cycling stability. This durability can be ascribed to the stabilizing role of boron moieties, which protect the carbon lattice from oxidative damage and preserve catalytic functionality.

The B-PW-BC catalyst retained high activity throughout, with only a slight decline (<10%) in overall removal efficiency after the fourth cycle. This minor reduction can be attributed to partial pore blockage or deposition of oxidation by-products.

These results confirm that boron doping not only enhances catalytic activity but also improves the structural stability of pyrochar under oxidative conditions, making it a robust and reusable carbocatalyst suitable for practical wastewater treatment applications.

### 3.7. Evaluation of Catalytic Performance on a Real Wastewater Sample

The efficiency of persulfate-based catalytic systems can be substantially influenced by constituents of real aqueous environments, such as inorganic anions (Cl^−^, SO_4_^2−^, H_2_PO_4_^−^), organic matter, and variations in pH [50]. These matrix components compete with both catalyst active sites and generated radical species, which often leads to lower performance compared to model solutions.

**Figure 11:**
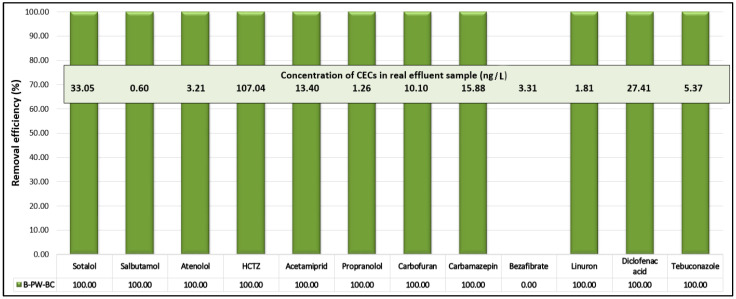
Removal efficiencies (%) of the identified CECs in the real effluent sample in the presence of B-doped pyrochar after 10 min of PSO (experimental conditions: [carbocatalyst] = 1 g/L; [PDS] = 5 mM; T = 25 °C; initial effluent sample pH = 7.9). An efficiency of 100% was achieved when the residual concentration of the compound of interest at the end of the process was below the quantification limit of the applied method.

To assess the practical applicability of B-doped pyrochar, catalytic tests were performed on a real wastewater effluent collected at the discharge point of a local WWTP into a nearby lake. The effluent quality complied with the Serbian Regulation on Emission Limit Values for Pollutants in Water [51]. Targeted UHPLC-MS/MS analysis (Figure 11) identified 12 CECs: four pesticides (acetamiprid, carbofuran, linuron, and tebuconazole) and eight PhACs (sotalol, salbutamol, atenolol, hydrochlorothiazide (HCTZ), propranolol, carbamazepine, bezafibrate, and diclofenac acid). Among them, HCTZ was detected at the highest concentration (107 ng/L), while all others were present at levels up to 35 ng/L.

Upon treatment with the B-PW-BC/PDS system, nearly complete removal (>95%) of all detected pollutants was achieved within 10 min, with the sole exception of bezafibrate, which remained largely unaffected. This resistance can be attributed to its specific molecular features: the presence of a chlorobenzene moiety introduces strong electron-withdrawing effects that lower the electron density of the aromatic ring, reducing its susceptibility to oxidative attack. In addition, the ionized carboxyl group at near-neutral pH increases hydration and weakens electrostatic interactions with the nearly neutral pyrochar surface (pH_ZC_ ≈ 7.1), thereby limiting adsorption. Since adsorption is a prerequisite for the hook-and-destroy mechanism, the reduced affinity of bezafibrate toward the catalyst surface prevents efficient catalytic activation. Furthermore, competition with organic matter and inorganic anions in the real wastewater matrix may have further inhibited its removal.

These findings highlight the excellent adaptability of the B-doped pyrochar under realistic water matrix conditions, confirming its potential as a practical and robust catalyst for wastewater remediation.

## 4. Conclusions

In this study, a sustainable and highly efficient B-doped pyrochar was prepared from pinewood sawdust via a simple one-step pyrolytic process and evaluated as a metal-free bifunctional catalyst for the removal of 25 contaminants of emerging concern (CECs), including 15 pesticides and 10 pharmaceutically active compounds (PhACs).

The obtained material demonstrated outstanding adsorption capacity and superior catalytic performance in persulfate activation. Nearly complete removal of most target pollutants was achieved within minutes, with B-doped pyrochar significantly outperforming pristine, N-doped, and N,B-codoped analogs. The catalyst also exhibited excellent reusability, retaining high activity after four consecutive cycles with only minor efficiency losses for a few organophosphorus pesticides and one PhACs (atenolol).

Mechanistic investigations combining quenching experiments and surface characterization revealed the coexistence of radical (SO_4_•^−^, •OH, O_2_•^−^) and non-radical (^1^O_2_, electron transfer pathway, ETP) mechanisms. While organophosphorus pesticides were degraded through combined radical and non-radical pathways, the removal of most other investigated CECs proceeded predominantly via non-radical ETP, facilitated by π–π interactions, hydrogen bonding, and Lewis acid sites introduced by boron doping. This synergy underpins the efficiency of the hook-and-destroy strategy, in which pollutants are first adsorbed and subsequently oxidized in situ.

Finally, evaluation on real wastewater effluent confirmed the practical adaptability of the B-doped pyrochar/PDS system, achieving >95% removal of 11 out of 12 identified CECs within 10 min. Bezafibrate was the only compound not effectively removed, which can be explained by its strong electron-withdrawing chlorobenzene group, ionized carboxyl functionality, and poor adsorption affinity at neutral pH. These findings highlight B-doped pyrochar as a robust, cost-effective, and environmentally benign carbocatalyst with strong potential for advanced wastewater treatment applications.

## Figures and Tables

**Table 1:** Removal efficiencies (%) of selected pesticides in the presence of B-doped pyrochar after 10 min of adsorption, 10 min of pre-adsorption and 10 and 90 min of PSO, and 10 and 90 min of PSO (experimental conditions: [individual CEC] = 30 μg/L; [carbocatalyst] = 1 g/L; [PDS] = 5 mM; T = 25 °C; initial solution pH = 6.5).

Pesticide	Type and Duration of the Process	Removal Efficiency in the Adsorption Process (%)	Adsorption Efficiency in PSO (%)	Degradation Efficiency in PSO (%)
Methamidophos	AD 10 min	59.1		
	AD 10 min + PSO 10 min		2.4	91.1
	AD 10 min + PSO 90 min		1.4	97.8
	PSO 10 min		0.9	95.4
	PSO 90 min		0.3	99.7
Omethoate	AD 10 min	63.0		
	AD 10 min + PSO 10 min		38.8	31.8
	AD 10 min + PSO 90 min		33.7	21.3
	PSO 10 min		41.4	25.9
	PSO 90 min		32.6	22.0
Diazinon	AD 10 min	100 *		
	AD 10 min + PSO 10 min		35.1	65.0
	AD 10 min + PSO 90 min		15.6	84.4
	PSO 10 min		31.1	68.9
	PSO 90 min		15.7	84.4
Trichlorfon	AD 10 min	100		
	AD 10 min + PSO 10 min		46.0	37.0
	AD 10 min + PSO 90 min		32.5	37.1
	PSO 10 min		41.7	37.7
	PSO 90 min		31.1	39.5
Dimethoate	AD 10 min	100		
	AD 10 min + PSO 10 min		28.3	71.7
	AD 10 min + PSO 90 min		26.6	73.4
	PSO 10 min		26.0	74.0
	PSO 90 min		24.6	75.5
Acetamiprid	AD 10 min	100		
	AD 10 min + PSO 10 min		26.5	73.5
	AD 10 min + PSO 90 min		27.0	73.0
	PSO 10 min		21.5	78.5
	PSO 90 min		20.8	79.2
Phosphamidon	AD 10 min	100		
	AD 10 min + PSO 10 min		56.4	43.6
	AD 10 min + PSO 90 min		57.0	43.0
	PSO 10 min		43.5	55.0
	PSO 90 min		40.6	59.4
Carbofuran	AD 10 min	100		
	AD 10 min + PSO 10 min		33.7	66.3
	AD 10 min + PSO 90 min		33.2	66.8
	PSO 10 min		23.9	76.1
	PSO 90 min		21.1	79.0
Imazalil	AD 10 min	100		
	AD 10 min + PSO 10 min		27.3	72.7
	AD 10 min + PSO 90 min		15.3	84.7
	PSO 10 min		12.5	87.5
	PSO 90 min		12.9	87.1
Methidathion	AD 10 min	100		
	AD 10 min + PSO 10 min		45.7	54.3
	AD 10 min + PSO 90 min		29.6	70.4
	PSO 10 min		39.5	60.6
	PSO 90 min		40.2	59.8
Ethoprophos	AD 10 min	100		
	AD 10 min + PSO 10 min		27.0	73.0
	AD 10 min + PSO 90 min		19.0	81.0
	PSO 10 min		23.6	76.5
	PSO 90 min		21.3	78.7
Carbaryl	AD 10 min	100		
	AD 10 min + PSO 10 min		14.4	85.6
	AD 10 min + PSO 90 min		12.1	87.9
	PSO 10 min		9.9	90.1
	PSO 90 min		12.2	87.8
Linuron	AD 10 min	100		
	AD 10 min + PSO 10 min		40.2	59.8
	AD 10 min + PSO 90 min		24.4	75.6
	PSO 10 min		31.4	68.6
	PSO 90 min		24.2	75.8
Malathion	AD 10 min	100		
	AD 10 min + PSO 10 min		31.0	69.0
	AD 10 min + PSO 90 min		16.3	83.7
	PSO 10 min		34.4	65.6
	PSO 90 min		0.0	100.0
Propiconazole	AD 10 min	100		
	AD 10 min + PSO 10 min		24.5	75.5
	AD 10 min + PSO 90 min		11.0	89.1
	PSO 10 min		48.0	52.1
	PSO 90 min		47.4	52.6

AD—Adsorption; PSO—Persulfate oxidation; * An efficiency of 100% was achieved when the residual concentration of the compound of interest at the end of the process was below the quantification limit of the applied method.

**Table 2:** Reusability of B-doped pyrochar/PDS system for the degradation of 15 selected pesticides and 10 PhACs after 10 min of PSO (experimental conditions: [individual CEC] = 30 μg/L; [carbocatalyst] = 1 g/L; [PDS] = 5 mM; T = 25 °C; initial solution pH = 6.5).

Pesticide	Removal Efficiency in 1st Cycle (%)	Removal Efficiency in 2nd Cycle (%)	Removal Efficiency in 3rd Cycle (%)	Removal Efficiency in 4th Cycle (%)
Methamidophos	100 *	100	98.8	98.2
Omethoate	49.7	36.5	41.6	39.0
Diazinon	100	100	100	100
Trichlorfon	70.3	63.9	65.9	62.9
Dimethoate	100	100	100	100
Acetamiprid	100	100	100	100
Phosphamidon	100	100	100	100
Carbofuran	100	100	100	100
Imazalil	100	100	100	100
Methidathion	100	100	100	100
Ethoprophos	100	100	100	100
Carbaryl	100	100	100	100
Linuron	100	100	100	100
Malathion	100	100	100	100
Propiconazole	100	100	100	100
**PhACs**				
Sotalol	100	100	100	100
Acetaminophen	100	100	100	100
Salbutamol	100	100	100	100
Atenolol	95.6	93.0	92.5	93.9
Famotidine	98.4	97.8	98.8	98.8
Ranitidine	99.9	99.7	100	100
Propranolol	100	100	100	100
HCTZ	100	100	100	100
Diltiazem	100	100	100	100
Carbamazepine	100	100	100	100

* An efficiency of 100% was achieved when the residual concentration of the compound of interest at the end of the process was below the quantification limit of the applied method.

## Data Availability

The original contributions presented in this study are included in the article/Supplementary Material. Further inquiries can be directed to the corresponding author.

## Supplementary Materials

The following supporting information can be downloaded at: https://www.mdpi.com/article/10.3390/toxics13121035/s1, Table S1: List of contaminants of emerging concern (CECs) included in the study with their chemical structure, compound class, CAS number, and method limit of quantification (MLOQ). Figure S1: HRSEM image of pristine pyrochar. Figure S2: XRD pattern of pristine pyrochar (References [52,53,54,55] are cited in the supplementary materials).
