# Supplementary material for: Hook-and-Destroy Strategy for Efficient Activation of Persulfate by B-Doped Pyrochar for the Removal of Contaminants of Emerging Concern from Wastewater
Source: Toxics. 2025 Nov 29;13(12):1035. doi: 10.3390/toxics13121035 (PMC12737626; doi:10.3390/toxics13121035)
Supplement: Supplementary file 1 [file toxics-13-01035-s001.zip › toxics-3974705-supplementary.pdf]

Table S1. List of contaminants of emerging concern (CECs) included in the study with their chemical structure, compound class, CAS number, and method limit of quantification (MLOQ)

CECs	Chemical structure[1]	Class[2,3]	CAS No.[4]	Method limit of quantification (µg/L)
**Methamidophos**	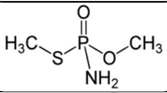	organophosphate insecticide	10265-92-6	0.21
**Omethoate**	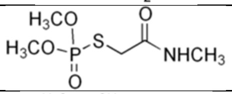	organophosphate insecticide	1113-02-6	0.10
**Diazinon**	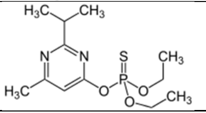	organophosphate insecticide	333-41-5	0.12
**Trichlorfon**	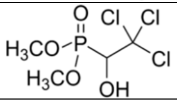	organophosphate insecticide	52-68-6	0.26
**Dimethoate**	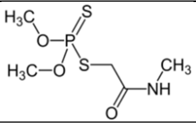	organophosphate insecticide	60-51-5	0.16
**Acetamiprid**	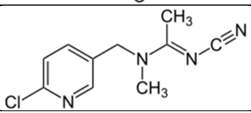	insecticide	135410-20-7	0.12
**Phosphamidon**	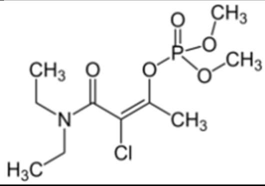	organophosphate insecticide	13171-21-6	0.17
**Carbofuran**	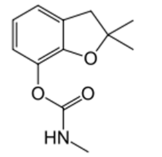	insecticide and nematicide	1563-66-2	0.075
**Imazalil**	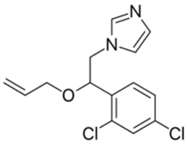	fungicide	35554-44-0	0.12
**Methidathion**	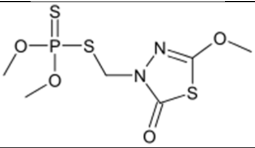	organophosphate insecticide	950-37-8	0.085
**Ethoprophos**	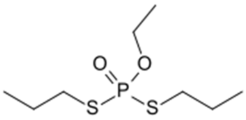	organophosphate insecticide	13194-48-4	0.13
**Carbaryl**	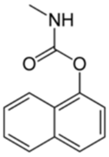	insecticide	63-25-2	0.17

**Linuron**	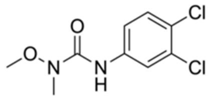	herbicide	330-55-2	0.17
**Malathion**	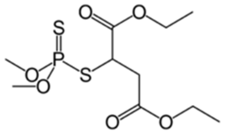	organophosphate insecticide	121-75-5	0.22
**Propiconazole**	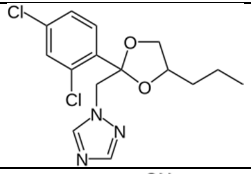	fungicide	60207-90-1	0.85
**Sotalol**	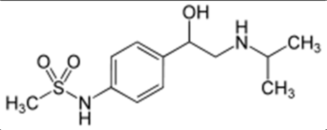	beta-blockers	3930-20-9	0.22
**Acetaminophen**	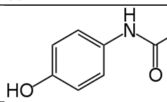	analgesics / antipyretics	103-90-2	0.12
**Salbutamol**	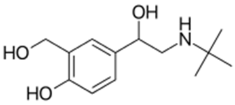	respiratory drugs	18559-94-9	0.13
**Atenolol**	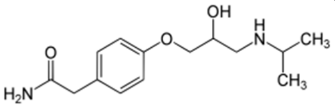	beta-blockers	29122-68-7	0.10
**Famotidine**	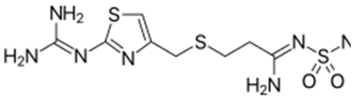	gastrointestinal drugs	76824-35-6	0.17
**Ranitidine**	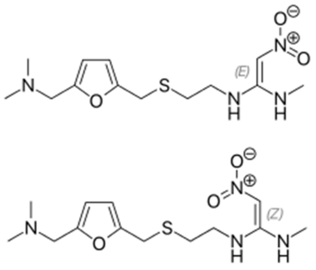	gastrointestinal drugs	66357-35-5	0.11
**Propranolol**	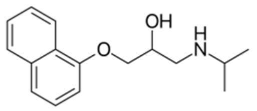	beta-blockers	525-66-6	0.11
**Hydrochlorothiazide (HTCZ)**	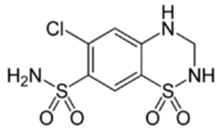	diuretics	58-93-5	0.12
**Diltiazem**	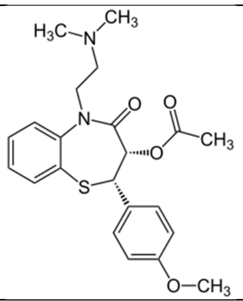	calcium channel blockers (CCBs)	42399-41-7	0.12

**Carbamazepine**	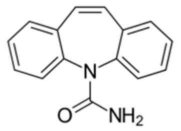	antiepileptic drug (AED)	298-46-4	0.095

[1] ChemSpider [52]; [2] EU Pesticides Database [53]; [3] Drugs.com [54]; [4] PubChem [55].

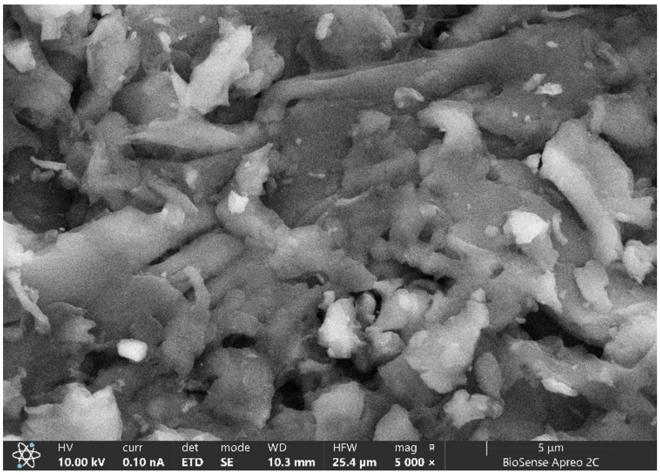

Figure S1. HRSEM image of pristine pyrochar.

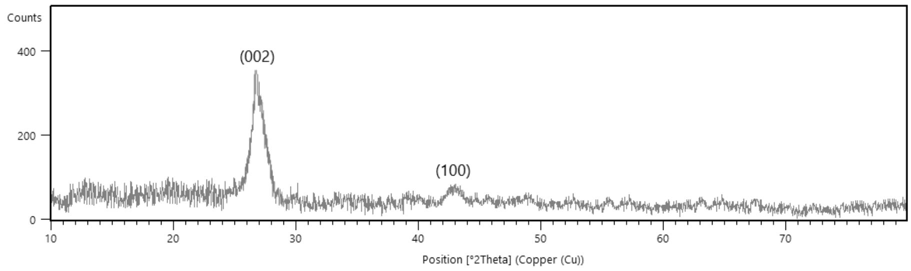

Figure S2. XRD pattern of pristine pyrochar.
